# Level I PD‐MCI Using Global Cognitive Tests and the Risk for Parkinson's Disease Dementia

**DOI:** 10.1002/mdc3.13451

**Published:** 2022-04-29

**Authors:** Judith A. Boel, Rob M.A. de Bie, Ben A. Schmand, John C. Dalrymple‐Alford, Connie Marras, Charles H. Adler, Jennifer G. Goldman, Alexander I. Tröster, David J. Burn, Irene Litvan, Gert J. Geurtsen, MDS Study Group Mild Cognitive Impairment in Parkinson's Disease, Bryan Bernard, Glenn Stebbins, J. Vincent Filoteo, Daniel Weintraub, John N. Caviness, Christine Belden, Cyrus P. Zabetian, Brenna A. Cholerton, Xuemei Huang, Paul J. Eslinger, James B. Leverenz, Sarah Duff‐Canning, Matt Farrer, Tim J. Anderson, Daniel J. Myall, Sharon L. Naismith, Simon J.G. Lewis, Glenda M. Halliday, Ruey‐Meei Wu, Caroline H. Williams‐Gray, David P. Breen, Roger A. Barker, Alison J. Yarnall, Martin Klein, Brit Mollenhauer, Claudia Trenkwalder, Jaime Kulisevsky, Javier Pagonabarraga, Carmen Gasca‐Salas, Maria C. Rodriguez‐Oroz, Carme Junque, Barbara Segura, Paolo Barone, Gabriella Santangelo, Davide M. Cammisuli, Roberta Biundo, Angelo Antonini, Luca Weis, Kenn Freddy Pedersen, Guido Alves

**Affiliations:** ^1^ Department of Neurology Amsterdam UMC Location University of Amsterdam Amsterdam The Netherlands; ^2^ Department of Psychology University of Amsterdam Amsterdam The Netherlands; ^3^ Amsterdam Neuroscience Amsterdam The Netherlands; ^4^ Department of Medical Psychology Amsterdam UMC Location University of Amsterdam Amsterdam The Netherlands; ^5^ New Zealand Brain Research Institute and School of Psychology, Speech and Hearing University of Canterbury Christchurch New Zealand; ^6^ Morton and Gloria Shulman Movement Disorders Centre and the Edmond J Safra Program in Parkinson's Disease, Toronto Western Hospital University of Toronto Toronto Canada; ^7^ Arizona Study of Aging and Neurodegenerative Disorders, Mayo Clinic Arizona, Scottsdale, Arizona, USA and Banner Sun Health Research Institute Sun City Arizona USA; ^8^ Department of Physical Medicine and Rehabilitation and Neurology Shirley Ryan AbilityLab and Northwestern University Chicago Illinois USA; ^9^ Department of Clinical Neuropsychology and Center for Neuromodulation Barrow Neurological Institute Phoenix Arizona USA; ^10^ Institute of Neuroscience Newcastle University Newcastle upon Tyne UK; ^11^ Parkinson and Other Movement Disorder Center, Department of Neurosciences University of California San Diego California USA

**Keywords:** dementia, mild cognitive impairment, global cognitive tests, Parkinson's disease, diagnostic accuracy

## Abstract

**Background:**

The criteria for PD‐MCI allow the use of global cognitive tests. Their predictive value for conversion from PD‐MCI to PDD, especially compared to comprehensive neuropsychological assessment, is unknown.

**Methods:**

The MDS PD‐MCI Study Group combined four datasets containing global cognitive tests as well as a comprehensive neuropsychological assessment to define PD‐MCI (n = 467). Risk for developing PDD was examined using a Cox model. Global cognitive tests were compared to neuropsychological test batteries (Level I&II) in determining risk for PDD.

**Results:**

PD‐MCI based on a global cognitive test (MMSE or MoCA) increases the hazard for developing PDD (respectively HR = 2.57, *P* = 0.001; HR = 4.14, *P* = <0.001). The C‐statistics for MMSE (0.72) and MoCA (0.70) were lower than those based on neuropsychological tests (Level I = 0.82; Level II = 0.81). Sensitivity, specificity and diagnostic accuracy balance was best in Level II.

**Conclusion:**

MMSE and MoCA predict conversion to PDD. However, Level II neuropsychological assessment seems the preferred assessment for PD‐MCI.

The MDS PD Mild Cognitive Impairment (PD‐MCI) diagnostic criteria^1^ operationalize two levels of cognitive assessment. Level I assessment is based on a global cognitive test (Level I‐GCT) or an abbreviated neuropsychological assessment (Level I‐NPA); and Level II is based on a comprehensive neuropsychological assessment. The use of a global cognitive test has some practical advantages over both Level I‐NPA and Level II in terms of time, ease of administration, and costs. However, the diagnostic accuracy for current cognitive status (i.e. PD‐MCI) when using global cognitive tests is low compared to Level I‐NPA and Level II criteria.^2^ The predictive value of global cognitive tests for progression from PD‐MCI to Parkinson Disease Dementia (PDD) needs to be determined.

Prior analyses using Level I‐NPA and Level II methods showed that PD‐MCI diagnosed in these ways increases the hazard of PDD and aids in the prediction of PDD (Level I‐NPA Hazard Ratio (HR) 2.02–11.25; Level II HR 2.69–14.10: depending on cut‐off used).^3^, ^4^ This study investigates the predictive value of Level I‐GCT, i.e. the Mini‐Mental State Examination (MMSE) and Montreal Cognitive Assessment (MoCA), for developing PDD. In addition, the predictive value of global cognitive tests will be compared to Level I‐NPA and Level II. This will help to make a substantiated choice on how to assess PD‐MCI in research and clinical practice.

## Methods

This study is based on data from the MDS Study Group Mild Cognitive Impairment in Parkinson's Disease.^5^ The methods are briefly described below (see publications for additional details).^3^, ^4^

### Data Inclusion

This retrospective study combined four datasets containing global cognitive tests and additional data suitable for both Level I and Level II PD‐MCI assessment based on neuropsychological tests. Individual studies were included if they allowed Level II PD‐MCI assessment (i.e. at least two neuropsychological tests in each of the five cognitive domains at baseline^1^), included ≥75 patients at baseline, had follow‐up on PDD status for ≥67% of the baseline population and had used the MMSE and/or the MoCA to assess the global cognitive functioning. Four studies were included.^2^, ^6^, ^7^, ^8^ Supplementary Figure S1 displays the inclusion flowchart and Supplementary Table S1 provides cohort details, including PDD criteria. Demographic and clinical data were collected. MDS‐Unified Parkinson's disease rating scale (UPDRS)^9^ scores were converted to have a uniform measure of UPDRS‐III,^3^ subsequently referred to as UPDRS‐III.

### Application of the PD‐MCI Criteria

To determine PD‐MCI by global cognitive tests, a cut‐off of <26 for the MoCA^10^ and < 29 for the MMSE^11^ were used considering these match prevailing cut‐offs for PD‐MCI used in PD research and practice. Measures for subjective cognitive decline varied between studies and are described in Supplementary Table S1. A measure for functional independence at baseline was not included as PDD was an exclusion criterion.

In short, for the neuropsychological assessment (NPA), either one test per cognitive domain (Level I‐NPA) or two tests per cognitive domain (Level II) were used. PD‐MCI based on the NPA was defined as scores −1.5SD below the normative data for at least two tests (respectively out of five or ten).^3^, ^4^

### Statistics

Multiple imputation (MI) was used to account for incomplete data. Cox proportional hazards models were used to evaluate whether Level I‐CGT PD‐MCI at baseline compared to no PD‐MCI adds to the risk of PDD as estimated by age, gender, level of education, disease duration, UPDRS‐III, and depression. Time was measured from PD symptom onset until PDD or censoring.

To compare various operationalizations of the criteria, C‐statistics (bootstrap‐corrected) which indicate discriminative ability between models as a measure of goodness‐of‐fit were calculated. PDD risk factors, like time since symptom onset, were taken into consideration. Two patients who develop PDD can be ordered by their time to event (PDD). If the model rightly predicts a shorter time to PDD for the one who developed PDD first, the C‐statistic increases. Two patients, one of which develops PDD, can be ordered as well. If the model rightly predicts a shorter time to PDD for the one who developed PDD, the C statistic also increases.

In addition, at baseline we determined sensitivity, specificity, positive predictive value (PPV), negative predictive value (NPV) and diagnostic accuracy (DA) for PDD. This was done for Level I‐CGT as well as for Level I‐NPA and Level II PD‐MCI assessment.

## Results

A total of 467 patients were included (Table 1). Sixty‐nine patients (14.3%) developed PDD during follow‐up (range 0.5–9 years).

**TABLE 1:** Baseline characteristics

	N = 467
Age, years (mean, SD)	68.7 (8.8)
Gender, male (frequency, %)	293 (62.7)
Education, years (mean, SD)	14.0 (3.1)
MMSE (median, IQR)	28.0 (27–29)
MoCA (median, IQR)	25.0 (23–28)
PD symptom duration, years (median, IQR)	4.0 (2.0–8.0)
UPDRS III (median, IQR)	20 (13–28)

IQR, InterQuartile Range.

### Frequencies of PD‐MCI

MMSE scores were available in all four datasets; MoCA scores were available in three datasets. A total of 172 out of 467 (36.8%) patients fulfilled the criteria for PD‐MCI based on the MMSE. A total of 111 out of 365 (30.4%) patients fulfilled the criteria for PD‐MCI based on the MoCA. Applying Level II resulted in 120/467 PD‐MCI patients (25.7%), while level I‐NPA based on 5 tests resulted in 46/467 PD‐MCI patients (9.9%). Compared to Level I NPA, both Level II and Level I‐GCT identified more PD‐MCI cases.

### Predictive Value

The Cox proportional hazards models indicated a significant contribution of PD‐MCI as defined by MMSE and MoCA cutoff scores to the hazard of PDD (HRs respectively 2.57 and 4.14; see Supplementary Table 2). Age as well as UPDRS scores were significant contributors in the model including the MMSE but not in the model including the MoCA.

### Comparison of PD‐MCI Assessment Methods

The sensitivity and specificity were respectively: 67.7 and 68.1% for Level I‐GCT based on the MMSE; 57.1 and 75.9% for Level I‐CGT based on the MoCA; 32.8 and 94.7% for Level I‐NPA; and 66.7 and 80.0% for level II (see Table 2). The Diagnostic Accuracy (DA) was sufficient for the MMSE (67.7), good for MoCA (73.3) and Level II (78.4) and very good for Level I‐NPA (86.1). The results in the three datasets with both MMSE and MoCA were comparable. The MMSE has sufficient but limited sensitivity, specificity and DA. Level I‐NPA and MoCA have a low sensitivity and miss many cases. Specificity of the Level I‐NPA is very high resulting in a very good DA. Level II seems to have the most optimal balance of sensitivity and specificity and a good DA.

**TABLE 2:** Sensitivity, specificity, NPV, PPV and diagnostic accuracy

	MMSE	MoCA	Level I‐NPA	Level II
Sensitivity	67.7	57.1	32.8	66.7
Specificity	68.1	75.9	94.7	80.0
PPV	27.7	25.7	50.0	31.9
NPV	91.6	91.9	89.8	94.5
Diagnostic Accuracy	67.6	73.3	86.1	78.4

PPV, Positive Predictive Value; NPV, Negative Predictive Value.

The C‐statistics were as follows: 0.72 for Level I‐CGT based on the MMSE; 0.70 for Level I‐CGT based on the MoCA; 0.82 for Level I‐NPA^4^; and 0.81 for level II.^4^ The lower C‐statistics for MMSE and MoCA indicate lower added value to the risk for PDD in comparison to level I‐NPA and level II PD‐MCI.

## Discussion

Our results show that PD‐MCI based on the global cognitive tests MMSE and MoCA have predictive value for PDD. However, given the higher hazard ratios of Level I‐NPA and Level II^3^, ^4^ the predictive value of MMSE and MoCA are lower. The findings were corrected for demographic and clinical characteristics known to contribute to the hazard for PDD (age, sex, UDPRS III score). The results are in line with previous studies indicating PD‐MCI based on neuropsychological assessment to be a risk factor for PDD.^4^, ^12^ The significantly higher hazard ratio's for the development of PDD for PD‐MCI patients compared to patients with normal cognition concurs with the concept of MCI as a transitional stage between normal cognition and PDD.

No difference was found in the predictive value when comparing the MMSE and the MoCA. However, the C‐statistic is not well suited to pick up small differences between models and comparisons based on this statistic should be interpreted with caution. The reason we find little difference in PDD prediction between MMSE and MOCA in contrast to other mostly smaller studies is unclear. The size of the studies as well as the variation in cut‐off points used for PD‐MCI and PDD could have been of influence.^13^ In a study including 132 patients Hoops et al. (2009)^13^ found the % correctly diagnosed based on MMSE and MoCA to be equal as well, when using the same cut‐offs. They advised using MoCA due to the ceiling effect on the MMSE.^13^

The MMSE and MoCA resulted in the higher percentage of patients classified as PD‐MCI (36.8 and 30.4% respectively) than did the neuropsychological assessment (9.9% for level I‐NPA; 25.7% for Level II). Global cognitive tests seemed to identify PD‐MCI cases who do not develop PDD, possibly “false positive,” leading to lower C‐statistics as well as a lower balance in sensitivity and specificity. On the other hand, Level I‐NPA seems to be too limited and strict, as scores on 2 out of 5 tests need to be below the cut‐off, and therefore may miss patients who actually have PD‐MCI.

Overall we determined the sensitivity, specificity and DA in the same patients. For the MMSE these values are sufficient but limited, which corresponds with Hoops et al. (2009).^13^ The sensitivity of Level I‐NPA and MoCA is lower indicating that many cases are missed. However, the specificity of the Level I‐NPA is very high resulting in a good DA. Level II seems to have the most optimal balance of sensitivity and specificity and has a good DA, thus indicating that a Level II neuropsychological assessment is preferred over the global cognitive tests.

Strengths of our study include the use of a large multicenter, international sample, uniform application of the MDS PD‐MCI criteria, and direct comparison of the different prevailing operationalizations of PD‐MCI. Furthermore, while the relation between PD‐MCI, demographic and clinical characteristics, and PDD has been reported separately in previous studies,^12^, ^14^ the current study analyzed the effects jointly in the predictive models. Limitations included different methods between cohorts for patient recruitment, neuropsychological assessment, assessment of motor signs and clinical PDD criteria. The length of follow‐up of the studies included differed. Therefore, the PDD frequency varies. This potentially could influence the determination of the sensitivity, specificity and diagnostic accuracy. However, as the analyses of sensitivity, specificity and diagnostic accuracy were performed in the same groups comparability seems adequate. When using a one point lower cut‐off the % correctly diagnosed in Level I‐CGT hardly changed in a mixed PD‐MCI and PDD sample.^13^

In conclusion, PD‐MCI assessed by global cognitive tests increases the hazard ratio for the development of PDD after taking age, sex, education, PD motor symptom severity, and depression into account. This finding supports PD‐MCI being a risk factor for PDD. PD‐MCI assessed by neuropsychological assessment (both Level I and Level II) had higher predictive value over PD‐MCI assessed by MMSE or MoCA. Given better balance of sensitivity and specificity and Diagnostic Accuracy compared to the use of global cognitive tests and Level I neuropsychological assessment, Level II neuropsychological assessment seems the optimal method for detecting PD‐MCI.

## Author Roles

(1) Research project: A. Conception, B. Organization, C. Execution; (2) Statistical Analysis: A. Design, B. Execution, C. Review and Critique; (3) Manuscript: A. Writing of the first draft, B. Review and Critique.

JAB: 1A, 1B, 1C, 2A, 2B, 2C, 2A, 2B, 2C.

RMAB: 1A, 3A, 3B.

BAS: 1A, 1B, 2C, 3A, 3B.

JDA: 1A, 1B, 3B.

CM: 1A, 3B.

CHA: 1A, 3B.

JGG: 1A, 3B.

AIT: 1A, 3B.

DJB: 1A, 3B.

IL: 1A, 3B.

GJG: 1A, 1B, 1C, 2A, 2C, 3A, 3B.

## Disclosures

**Ethical Compliance Statement:** The Medical Ethics Committee of the University of Amsterdam stated that the approval of an institutional review board was not required for this work (reference number W12_3–1 # 13.17.0003). Each study site obtained informed consent obtained. We confirm that we have read the Journal's position on issues involved in ethical publication and affirm that this work is consistent with those guidelines.

**Funding Sources and Conflicts of Interest:** Funding was received from the Michael J. Fox Foundation. The authors have no conflicts of Interest for this study.

**Financial Disclosures for the Previous 12 Months:** Judith A. Boel, Rob M.A. de Bie, Ben A. Schmand, Jennifer G. Goldman, Alexander I. Tröster, David J. Burn, and Gert J. Geurtsen have no disclosures to report. John C. Dalrymple‐Alford, None, only grants from Tertiary Education Commission, New Zealand, Health Research Council of New Zealand, Canterbury Medical Research Foundation and Neurological Foundation of New Zealand. Connie Marras is a consultant for Gray Matter Technologies and receives financial compensation as a steering committee member from the Michael J Fox Foundation. She received grants from The Michael J Fox Foundation, Canadian Institutes of Health Research, Parkinson's Foundation (US), International Parkinson and Movement Disorders Society, Weston Brain Institute, Theravance Inc, Centogene. Charles H. Adler has Stock Ownership in medically‐related fields: Cionic; Consultancies: Avion, CND Life Science, Jazz Pharm, Neurocrine; Grants: NIH, Michael J. Fox Foundation, Arizona Biomedical Research Commission. Irene Litvan's research is supported by the National Institutes of Health grants: 2R01AG038791‐06A, U01NS100610, U01NS80818, R25NS098999; U19 AG063911‐1 and 1R21NS114764‐01A1; the Michael J Fox Foundation, Parkinson Foundation, Lewy Body Association, CurePSP, Roche, Abbvie, Biogen, Centogene. EIP‐Pharma, Biohaven Pharmaceuticals, Novartis, Brain Neurotherapy Bio and United Biopharma SRL—UCB. She was a member of the Scientific Advisory Board of Lundbeck and is a Scientific advisor for Amydis. She receives her salary from the University of California San Diego and as Chief Editor of *Frontiers in Neurology*.

## Supporting information


**Supplementary Figure S1**. Flowchart showing the data inclusion process
**Supplementary Table S1**. Cohort details of the included studies.
**Supplementary Table S2**. Hazard ratios of models containing MMSE and MoCA.

**Supplementary Text S1**. Members of the International Parkinson and Movement Disorders Society Mild Cognitive Impairment (MCI) Study Group.
