# Supplementary material for: Level I PD‐MCI Using Global Cognitive Tests and the Risk for Parkinson's Disease Dementia
Source: Mov Disord Clin Pract. 2022 Apr 29;9(4):479–83. doi: 10.1002/mdc3.13451 (PMC9092740; doi:10.1002/mdc3.13451)
Supplement: Supplementary file 1 — Supplementary Figure S1. Flowchart showing the data inclusion process Supplementary Table S1. Cohort details of the included studies. Supplementary Table S2. Hazard ratios of models containing MMSE and MoCA. [file MDC3-9-479-s002.docx]

**Supplementary figure 1.** Flowchart showing the data inclusion process

Studies assessed for eligibility (n=24)

Excluded studies (n=20)

 Cross-sectional (n=7)

 Not conform Level II MDS PD-MCI (n=10)

 < 75 patients at baseline or < 67% patients at follow up (n=3)

Number of patients included in these studies (n=592)

Excluded patients (n=125)

 PDD diagnosis at first measurement (n=40)

 Disease duration > 25 years at first

measurement (n=3)

 Patients without follow-up (n=82)

Eligible studies (n=4)

Patients included in final

analysis (n = 467)

Missing Values

The percentage of missing values in age, sex, years of education, and PD symptom duration ranged from 0% to 1%. Gradual cognitive decline had 2% missing values, the UPDRS-III 6%, and depression-indicators were missing in 9%. One percent had a missing attention test, 2% a visuospatial and executive function respectively, 10% a memory, and 16% a language test. PD-MCI depends on multiple measures and was missing in 22%. Because only a small portion of the neuropsychological tests is missing and their mutual relations are strong, the conditions are such that multiple imputation is expected to perform well.

**Supplementary table 1.** Cohort details of the included studies.

| Cohort | CARPA (n = 112)^1^ | NZBRI (n = 136)^2^ | AZSAND (n = 101)^3^ | Toronto (n = 118)^4^ |
| --- | --- | --- | --- | --- |
| Cohort type | Closed incident clinic cohort | Closed prevalent clinic cohort | Open community volunteers cohort | Closed prevalent clinic cohort |
| PD criteria | Gelb | UKPDS Brain Bank | UKPDS Brain Bank | UKPDS Brain Bank |
| PDD criteria | Modified Dubois^*^ | MDS PDD | MDS PDD and  DSM-IV | MDS PDD |
| Normative scores | Published norms | Published  norms | Control group | Published norms |

| Subjective cognitive decline | Patient | PDQL item 31  and 34 | PDQ-39 item 32 &  CDR memory items |  | Abbreviated NBI patient version |
| --- | --- | --- | --- | --- | --- |
|  | Significant other |  | CDR memory items |  | Abbreviated NBI caregiver version |
|  | Clinician |  |  | UPDRS I item |  |
| Global cognitive test |  | MMSE | MMSE and MoCA | MMSE and MoCA | MMSE and MoCA |

Supplementary Table 1 shows the cohort types, diagnostic criteria, norms used for evaluation of neuropsychological performance and the measures of subjective cognitive decline for each of the studies. Abbreviations: CDR = Clinical Dementia Rating; NBI = Neurobehavioral Signs and Symptoms Abbreviated Inventory; UPDRS = Unified Parkinson's Disease Rating Scale; PDQ-39 = Parkinson's Disease Questionnaire 39; PDQL = Parkinson's Disease Quality of Life Questionnaire.

Supplementary References

1. Muslimović D, Post B, Speelman J, Schmand B. Cognitive profile of patients with newly diagnosed Parkinson disease. Neurology 2005;65:1239–1245.
2. Dalrymple-Alford JC, Livingston L, MacAskill MR, et al. Characterizing mild cognitive impairment in Parkinson's disease. Mov Disord 2011;26:629-636.
3. Beach T, Adler C, Sue L. Arizona Study of Aging and Neurodegenerative Disorders and Brain and Body Donation Program. Neuropathology 2015; 35, 354–389.
4. Marras C, Armstrong MJ, Meaney CA, et al. Measuring mild cognitive impairment in patients with Parkinson's disease. Mov Disord 2013;28:626-633.

**Supplementary table 2.** Hazard ratios of models containing MMSE and MoCA.

| **MMSE** *<29 for PD-MCI* | ***β*** | ***SE*** | ***95% CI*** | ***HR (eβ)*** | ***z-statistic*** | ***p*** |
| --- | --- | --- | --- | --- | --- | --- |
| Age (years) | 0.07 | 0.02 | 0.03 to 0.10 | 1.07 | 3.50 | <0.001 |
| Gender (male) | 0.32 | 0.28 | -0.23 to 0.88 | 1.38 | 1.13 | 0.26 |
| Education (years) | -0.01 | 0.05 | -0.11 to 0.09 | 0.99 | -0.24 | 0.81 |
| Depression indicator | 0.44 | 0.42 | -0.38 to 1.25 | 1.55 | 1.05 | 0.29 |
| PD-MCI (MMSE) | 0.94 | 0.30 | 0.36 to 1.52 | 2.57 | 3.19 | 0.001 |
| UPDRS-III | 0.42 | 0.18 | 0.07 to 0.78 | 1.53 | 2.32 | 0.02 |
| **MoCA** *<26 for PD-MCI* | ***β*** | ***SE*** | ***95% CI*** | ***HR (eβ)*** | ***z-statistic*** | ***p*** |
| Age (years) | 0.05 | 0.03 | -0.001 to 0.10 | 1.05 | 1.92 | 0.055 |
| Gender (male) | 0.11 | 0.36 | -0.60 to 0.83 | 1.12 | 0.30 | 0.76 |
| Education (years) | -0.08 | 0.07 | -0.21 to 0.05 | 0.92 | -1.26 | 0.21 |
| Depression indicator | -0.15 | 0.63 | -1.38 to 1.09 | 0.86 | -0.23 | 0.82 |
| PD-MCI (MoCA) | 1.42 | 0.36 | 0.71 to 2.12 | 4.14 | 3.95 | <0.001 |
| UPDRS-III | 1.15 | 0.72 | -0.28 to 2.56 | 3.16 | 1.59 | 0.11 |
