# Supplementary material for: Level I PD‐MCI Using Global Cognitive Tests and the Risk for Parkinson's Disease Dementia
Source: Mov Disord Clin Pract. 2022 Apr 29;9(4):479–83. doi: 10.1002/mdc3.13451 (PMC9092740; doi:10.1002/mdc3.13451)
Supplement: Supplementary file 2 — Supplementary Text S1. Members of the International Parkinson and Movement Disorders Society Mild Cognitive Impairment (MCI) Study Group. [file MDC3-9-479-s001.docx]

**Supplementary Text 1. This study was conducted on behalf of the International Parkinson and Movement Disorders Society Mild Cognitive Impairment (MCI) Study Group, consisting of the byline authors as well as the members listed here.**

Bryan Bernard PhD and Glenn Stebbins PhD (Department of Neurological Sciences, Section of Parkinson Disease and Movement Disorders, Rush University Medical Center, Chicago, IL);

J. Vincent Filoteo PhD (Department of Neurosciences University of California San Diego, Parkinson and Other Movement Disorder Center, San Diego, California );

Daniel Weintraub PhD (Departments of Psychiatry and Neurology and Parkinson’s Disease and Mental Illness Research, Philadelphia Veterans Affairs Medical Center, Philadelphia, PA);

John N. Caviness MD and Christine Belden PhD(Arizona Study of Aging and Neurodegenerative Disorders, Mayo Clinic Arizona, Scottsdale, AZ and Banner Sun Health Research Institute, Sun City, AZ);

Cyrus P. Zabetian MD and Brenna A. Cholerton PhD (Veterans Affairs Puget Sound Health Care System and Department of Neurology, University of Washington School of Medicine, Seattle, WA);

Xuemei Huang PhD and Paul J. Eslinger PhD (Department of Neurology, Hershey Medical Center);

James B. Leverenz MD (Lou Ruvo Center for Brain Health, Neurological Institute, Cleveland Clinic);

Sarah Duff-Canning PhD (Morton and Gloria Shulman Movement Disorders Clinic and the Edmond J Safra Program in Parkinson’s disease, Toronto Western Hospital, University of Toronto);

Matt Farrer PhD (The Centre for Applied Neurogenetics, University of British Columbia);

Tim J. Anderson FRACP and Daniel J Myall PhD (New Zealand Brain Research Institute, Brain Research New Zealand - Rangahau Roro Aotearoa, Christchurch);

Sharon L. Naismith PhD and Simon JG Lewis MD (Brain & Mind Centre, University of Sydney);

Glenda M. Halliday PhD (Brain & Mind Centre, University of Sydney and Neuroscience Research Australia, University of New South Wales);

Ruey-Meei Wu MD PhD (Department of Neurology, National Taiwan University Hospital, College of Medicine, National Taiwan University, Taipei, Taiwan);

Caroline H. Williams-Gray MRCP PhD, David P. Breen MRCP PhD and Roger A. Barker MRCP PhD (John Van Geest Centre for Brain Repair, University of Cambridge, Cambridge, UK );

Alison J. Yarnall MRCP PhD (Faculty of Medical Sciences, Newcastle University, Newcastle upon Tyne, UK);

Martin Klein PhD (Department of Medical Psychology, section Medical Neuropsychology, VU medical center, Amsterdam);

Brit Mollenhauer MD (Paracelsus-Elena-Klinik, Kassel, Germany, and University Medical Center Goettingen, Department of Neurosurgery and Institute of Neuropathology, Goettingen, Germany);

Claudia Trenkwalder MD (Paracelsus-Elena-Klinik, Kassel, Germany, and University Medical Center Goettingen, Department of Neurosurgery, Goettingen, Germany);

Jaime Kulisevsky MD PhD (Movement Disorders Unit, Neurology Department, Hospital and Institute of Biomedical Research Sant Pau, 'CIBERNED', Barcelona, Spain and 'Universitat Oberta de Catalunya');

Javier Pagonabarraga MD PhD (Movement Disorders Unit, Neurology Department, Hospital and Institute of Biomedical Research Sant Pau, 'CIBERNED', Barcelona, Spain);

Carmen Gasca-Salas MD PhD (Department of Neurology, Hospital Donostia, Donostia, San Sebastian and Ikerbasque, Basque Foundation for Science, Bilbao, Spain);

Maria C. Rodriguez-Oroz MD PhD (Clinica Universidad de Navarra Spain);

Carme Junque PhD (Department of Psychiatry and Clinical Psychobiology, Faculty of Medicine, IDIBAPS, University of Barcelona, Spain);

Barbara Segura PhD (Department of Psychiatry and Clinical Psychobiology, Faculty of Medicine, IDIBAPS, University of Barcelona, Spain);

Paolo Barone PhD (Neurogenerative disease centre, University of Salerno, Salerno, Italy);

Gabriella Santangelo PhD (Department of Psychology, Second University of Naples, Italy);

Davide M Cammisuli PhD (Department of Surgery, Medical, Molecular, and Critical Area Pathology, University School of Medicine, Pisa);

Roberta Biundo PhD, Angelo Antonini PhD and Luca Weis PhD (Parkinson Unit, Fondazione Ospedale San Camillo IRCCS, Venice);

Kenn Freddy Pedersen PhD and Guido Alves PhD (The Norwegian Centre for Movement Disorders, Department of Neurology, and Memory Clinic, Stavanger University Hospital, Stavanger, Norway).
